# Conjugative DNA Transfer Induces the Bacterial SOS Response and Promotes Antibiotic Resistance Development through Integron Activation

**DOI:** 10.1371/journal.pgen.1001165

**Published:** 2010-10-21

**Authors:** Zeynep Baharoglu, David Bikard, Didier Mazel

**Affiliations:** 1 Institut Pasteur, Unité Plasticité du Génome Bactérien, Département Génomes et Génétique, Paris, France; 2 CNRS, URA2171, Paris, France; Université Paris Descartes, INSERM U571, France

## Abstract

Conjugation is one mechanism for intra- and inter-species horizontal gene transfer among bacteria. Conjugative elements have been instrumental in many bacterial species to face the threat of antibiotics, by allowing them to evolve and adapt to these hostile conditions. Conjugative plasmids are transferred to plasmidless recipient cells as single-stranded DNA. We used *lacZ* and *gfp* fusions to address whether conjugation induces the SOS response and the integron integrase. The SOS response controls a series of genes responsible for DNA damage repair, which can lead to recombination and mutagenesis. In this manuscript, we show that conjugative transfer of ssDNA induces the bacterial SOS stress response, unless an anti-SOS factor is present to alleviate this response. We also show that integron integrases are up-regulated during this process, resulting in increased cassette rearrangements. Moreover, the data we obtained using broad and narrow host range plasmids strongly suggests that plasmid transfer, even abortive, can trigger chromosomal gene rearrangements and transcriptional switches in the recipient cell. Our results highlight the importance of environments concentrating disparate bacterial communities as reactors for extensive genetic adaptation of bacteria.

## Author Summary

Bacteria exchange DNA in their natural environments. The process called conjugation consists of DNA transfer by cell contact from one bacterium to another. Conjugative circular plasmids have been identified as shuttles and reservoirs for adaptive genes. It is now established that such lateral gene transfer plays an essential role, especially for the antibiotic resistance development and dissemination among bacteria. Moreover, integrons, platforms of mobile gene cassettes, have been instrumental in this phenomenon, through their successful association with conjugative resistance plasmids. We demonstrate in this study that the conjugative transfer of plasmids triggers a bacterial stress response—the SOS response—in recipient cells and can impact the cassette content of integrons. The SOS response is already known to induce various genome modifications. Human and animal pathogens cohabit with environmental bacteria, in niches which will favor DNA exchange. SOS induction during conjugation is thus most probably able to impact a wide range of genomes. Bacterial SOS response could then be a suitable target for co-treatment of infections in order to prevent exchange of antibiotic resistance/adaptation genes.

## Introduction

Free-living bacteria commonly face changing environments and must cope with varying conditions. These adaptive strategies involve temporary physiological responses through various groups of genes gathered in regulons that are induced or repressed according to the surrounding conditions. This is the case for the quorum sensing regulon [1], [2], the stringent response and catabolite repression systems, which allow adjustment of gene expression according to the growth conditions [3]–[5]. In other instances, the only adaptive solution requires a genetic change, and bacteria have developed mechanisms that favour genome modifications either by transiently increasing their mutation rates, inducing re-arrangements, or lateral (horizontal) gene transfer (HGT). One of the better known responses of this kind is the trigger of the SOS regulon, which controls DNA repair and recombination genes [6].

SOS is a bacterial stress response induced when an abnormal rate of single stranded DNA (ssDNA) is present in the cell. ssDNA is the substrate for RecA polymerization. The formation of a ssDNA/RecA nucleofilament stimulates auto-proteolysis of the LexA repressor, leading to de-repression of genes composing the SOS regulon. The SOS response is triggered by the accumulation of ssDNA, for example when cells try to replicate damaged DNA, after UV irradiation or treatment with antibiotics (fluoroquinolones, β-lactams) or mitomycin C (MMC), a DNA cross-linking agent. In addition to these endogenous sources, ssDNA is also produced by several mechanisms of exogenous DNA uptake involved in lateral gene transfer, namely by conjugation, transformation and occasionally transduction.

Conjugation is indeed one mechanism of lateral transfer that leads to the transient occurrence of ssDNA in the recipient cell [7], [8]. The presence of anti-SOS factors in some conjugative plasmids, such as the *psiB* gene of R64*drd* and R100-1 [9], suggests that conjugative DNA transfer can induce SOS. In plasmid R100-1, *psiB* (plasmidic SOS inhibition) was shown to be transiently expressed during the first 20 to 40 minutes of conjugation [10], [11] from a ssDNA promoter [12], and inhibited the bacterial SOS response [13], [14]. Plasmids carrying *psiB* do not all express it at levels sufficient to alleviate SOS, as seems to be the case in F plasmids for instance [9], [10], [13], [15], [16].

Conjugation is a widespread mechanism in the intestinal tract of host animals where there is a high concentration of bacterial populations [17]–[22]). Lateral gene transfer plays a large role in the evolution of genomes and emergence of new functions, such as antibiotic resistance, virulence and metabolic activities in bacterial species [23].

Bacteria can also possess other internal adaptive genetic resources. *Vibrio cholerae* carries a superintegron (SI), that can be used as a reservoir of silent genes that can be mobilized when needed. Integrons are natural gene expression systems allowing the integration of an ORF by site-specific recombination, transforming it into a functional gene [24]. Multi-resistant integrons (RI) have been isolated on mobile elements responsible for the assembly and rapid propagation of multiple antibiotic resistances in Gram-negative bacteria through association with conjugative plasmids [25], [26]. An integron is characterized by an *intI* gene, coding a site specific recombinase from the tyrosine recombinase family, and an adjacent primary recombination site *attI* [27]. The IntI integrase allows the integration of a circular promoterless gene cassette carrying a recombination site, *attC*, by driving recombination between *attI* and *attC* [28]. The integrated gene cassettes are expressed from the Pc promoter located upstream of the *attI* site in the integron platform [29]. The discovery of integrons in the chromosome of environmental strains of bacteria, and among these the superintegrons (SI) mentioned above, has led to the extension of their role from the “simple” acquisition of resistance genes to a wider role in the adaptation of bacteria to different environments [30].

The dynamics of cassette recombination and the regulation of integrase expression are poorly understood. Recently, it was shown that *intI* is regulated by the bacterial SOS response [31]. Since SOS is now known to induce both the RI and *V.cholerae* integrase expression, an important issue is to understand when and where cassette recombination takes place and how the integrase inducing SOS response is activated.

Our objective was to determine if conjugative ssDNA transfer can trigger the SOS response, and to which extent this affects *intI* expression and cassette recombination. SOS induction in promiscuous environments can prepare bacteria to face the many threats they can encounter there. In order to understand regulatory networks existing between conjugation and its effect on integron content and cassette expression, we first addressed if conjugation induces SOS using reporter fusions in *V. cholerae* and *E. coli*. After quantifying expression from the *V. cholerae intIA* promoter using GFP fusions, we adopted a genetic approach to test integrase-dependent site-specific recombination *in vivo*.

We show that conjugative plasmid transfer generally induces the SOS response and up-regulates integrase expression, triggering cassette recombination. However, this is not the case when an anti-SOS factor (*psiB*) is expressed, as seen for some narrow host range conjugative plasmids isolated from *Enterobacteria*. We further show that this anti-SOS function prevents up-regulation of the SOS regulon in a host-specific manner after conjugation. We demonstrate that conjugative transfer is sufficient to trigger integron cassette recombination in recipient cells. This study outlines the connections between conjugative lateral DNA transfer, bacterial stress response and recombination of gene cassettes in integrons, and provides new insights into the development of the antibiotic resistance within a population.

## Results

### Conjugative transfer of plasmids R388, R6K*drd*, and RP4 induces the SOS response in *E. coli* and *V. cholerae*

During conjugation, plasmid DNA enters the host cell in a single stranded fashion [7], [8]. In order to test whether conjugation induces the SOS response in the recipient cell, we used reporter *E. coli* and *V. cholerae* strains carrying *sfiA::lacZ* (7651) and *recN::lacZ* (7453) β-galactosidase fusions, respectively. *sfiA* (cell division in *E.coli*) and *recN* (recombinational repair) genes belong to the SOS regulon of *E.coli*. We also identified a LexA binding box upstream of *recN* in *V. cholerae*. We confirmed that induction of SOS in these strains results in expression of the β-galactosidase (β-gal) enzyme (not shown). Table 1 summarizes the conjugative plasmids belonging to several incompatibility groups we used in this study [32]. The donor (DH5α) strain was *recA-* and *ΔlacZ*.

**Table 1:** Conjugative plasmids used in this study.

Conjugative plasmid ; (incompatibility group)	*psiB* gene	SOS induction ; *E. coli/V. cholerae*
RP4 (IncP) ; ap km	No	+/+
R6K*drd* (IncX) ; ap	No	++/++
R388 (IncW) ; trim	No	++/++
R64*drd* (IncI1) ; tc	Yes	−/+++
R100-1 (IncFII) ; cm sm sp tc	Yes	−/+++
RIP113 (IncN) ; tc	Yes	−/+++
Rs-a (IncW) ; sm km cm	No	+/+

R388 and R64 carry an RI platform. Antibiotic resistances: ap: ampicillin; km: kanamycin; trim: trimethoprim; tc: tetracycline; cm: chloramphenicol; sm: streptomycin; sp: spectinomycin.

**Figure 1:**
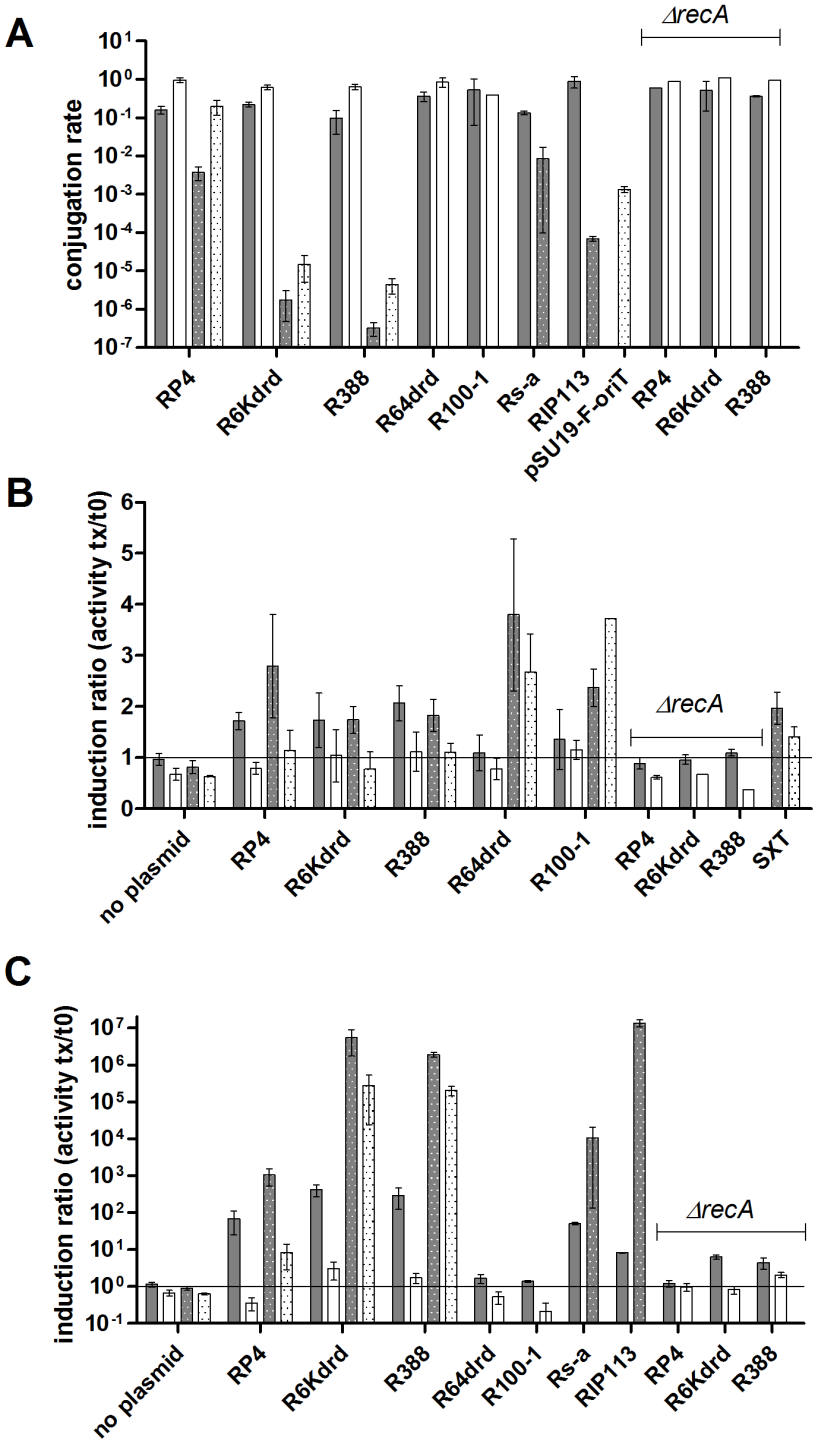
Conjugation induces SOS in *E. coli* and *V. cholerae*. Shaded bars: *E. coli*. Dotted bars: *V. cholerae*. Grey: values at peak of induction t_40_-t_60min_ (for SXT, the peak is shown at t_210_); White: values at t_240_. A: conjugation rates in *E. coli recA*+ and *recA* (strains 7651 and 7713) and *V. cholerae* (7453); B: SOS induction in total population of recipient *E. coli* and *V. cholerae* measured by β-gal tests; C: SOS induction ratio in conjugants only, for *E. coli* and *V. cholerae*. Induction was calculated as described in Materials and Methods. Induction ratios are units at time t_x_/units at time t_0_.

The conjugation rates of these plasmids were first measured at various time points after donor and recipient cells were mixed (Figure 1A). In *E. coli*, all plasmids conjugate approximately at the same rate so that nearly all recipients have received a plasmid after 60 min of conjugation. In *V. cholerae* transfer rates vary considerably, only 1 in 10^5^ cells have received a plasmid after 4h of mating with R6K*drd* and R388, while RP4 has a transfer rate similar to that of *E. coli* (10^−1^ to 1). Neither R64*drd* nor R100-1 replicate in *V. cholerae*. In order to address whether R100-1 actually transfers from *E. coli* into *V. cholerae*, we used pSU19-*oriT_F_* plasmids containing the *oriT_F_* (72 bp) of plasmid F. Plasmid F does not replicate in *V. cholerae* and *oriT_F_* is 98% identical to *oriT_R100_*. The high *oriT_F_* transfer rate observed at 1h of mating confirms that plasmids F and R100-1 (and presumably R64*drd*) can indeed transfer into *V. cholerae* and that the lack of R100/R64 transconjugants is due to their inability to establish themselves in this bacterium.

SOS induction linked to conjugation was measured in the total recipient population by counting the actual number of recipient cells plated on selective medium instead of using OD units (Materials and Methods), to obtained an induction value per potential recipient cell. Mating was interrupted at various time points (t_0_, t_40_, t_60_, t_120_, t_180_, t_240_) and β-gal activity was measured in both *E. coli* and *V. cholerae* recipients (Figure 1B). The results are represented on the graph as the induction ratios at times t_0_, t_60_ and t_240_ over the induction at t_0_. When the recipient strain was mixed with empty donor, no SOS induction was observed. A peak of SOS induction in *E .coli* was detected after 40 min to 60 min of mating with a conjugation proficient donor, 1.7 fold induction for RP4 and R6K*drd* and 2.3 fold for R388. The induction peak was also observed in *V. cholerae* (2.3 fold for R6K*drd*, 2.7 fold for RP4 and 3.4 fold for R388). To verify that the β-gal activity was due to the SOS induction, we deleted the *recA* gene in the recipient *E. coli* strain. No induction of β-gal activity was observed in the *ΔrecA* strain after conjugation with RP4, R6K*drd* and R388. This confirms that the β-gal induction observed in *recA+* strain indeed reflects the SOS induction by RP4, R6K*drd* and R388.

As described above, β-gal induction peaks between t_40_ and t_60_ minute of mating. The induction then decreases to reach the level shown at t_240_, forming bell shaped curves (data not shown). This induction pattern reflects the SOS induction in an asynchronous population of bacteria. It can be explained by the fact that plasmids RP4, R6K*drd* and R388 replicate in recipient cell. Once mating has started and as time goes by, there tends to be less plasmidless recipient cells. Indeed, entry of the plasmid DNA induces SOS, the incoming plasmidic ssDNA then replicates in the conjugant cell and the entry exclusion systems prevents entry of another plasmid [33], [34]. However, cells continue to divide so that the population of kanamycin resistant (kanR) host cells increases. Accordingly, even when the transfer rate remains constant (especially for low rated plasmids), the increase in the number of kanR cells can explain the drop of activity per recipient in the curve. The SOS response is expected to return to normal once all the cells have acquired the plasmid.

Since all cells in the recipient population have not received a conjugating DNA at the time of the β-gal assay, we calculated the SOS induction per conjugant, i.e. per recipient cell that has actually undergone DNA uptake (Materials and Methods). The results are represented as ratios over t_0_ in Figure 1C. As expected, the induction signal is amplified when one takes into account the conjugation rate for each plasmid. This amplification of several orders of magnitude is likely to be an effect of unsuccessful conjugation: SOS is induced by incoming DNA, that is not always converted into a replicating plasmid. The induction profiles, however, are compatible with Figure 1B: R388 and R6K*drd* strongly induce SOS, RP4 also shows a high induction, however it is lower than the former two plasmids. Once again, no (or very little) induction was observed in *E .coli ΔrecA* strain, confirming that conjugation induces RecA-dependent SOS response.

SOS induction by RP4 is weaker in both *E. coli* and *V. cholerae*, compared to induction during conjugation with R388 or R6K*drd*. We did not find any particular feature in the DNA sequence, or gene order of RP4 that could explain this observation. However, one can imagine that the higher transfer rate during RP4 conjugation is coupled with an early expression of entry exclusion systems, resulting in a quick decrease of ssDNA levels and repression of the SOS response.

To check if lower SOS induction was specific of RP4, we decided to test 2 other plasmids (Rs-a and RIP113) belonging to different incompatibility groups, at t_60_ - the peak for SOS induction observed for the plasmids mentioned earlier. Rs-a (IncW) induced SOS in *E. coli* and *V. cholerae* (Figure 1C and data not shown), confirming that SOS induction can be triggered by this plasmid. Interestingly, Rs-a conjugates at a rate of 10^−1^ and yields an intermediate induction level (like RP4). Even though we have a small sample of plasmids, SOS induction during mating seems to inversely correlate with conjugation rate (at 1h of mating) or replication of plasmids, except for R6K*drd*. Further study is needed to verify this observation. On the other hand, RIP113 (IncN) induced SOS in *V. cholerae* only (Figure S2).

An increasing number of non-replicative conjugative elements, generally named ICE, have been described in bacteria. One of the best studied is the SXT element discovered in *V. cholerae* [35]–[37]. We addressed if conjugative transfer of an SXT element integrated in the chromosome of *E. coli* [38] to *V. cholerae* also induces the SOS response. We observed a similar induction profile as for the conjugative plasmids with a peak of induction measured at t_210_ (Figure 1B). The delay can likely be explained by the very low transfer rate (10^−6^ after 6 six hours of mating).

Our results show that plasmids lacking the *psiB* gene (here RP4, R388, R6K*drd* and Rs-a) induce SOS upon conjugation into the recipient cell. On the other hand, RIP113 (IncN) induced SOS in *V. cholerae* only (Figure S2), thus behaving like R64*drd* and R100-1 plasmids.

### PsiB strongly alleviates the SOS response in *E. coli* but only very weakly in *V. cholerae*: conjugation with plasmids R64*drd* and R100-1 induces SOS in *V. cholerae*

R64*drd* and R100-1 plasmids do not induce (or very poorly) the SOS response in *E. coli* (Figure 1B and 1C). This was expected as these plasmids carry a *psiB* anti-SOS gene. Plasmid RIP113 behaves like R64*drd* and R100-1 in terms of SOS induction in *E. coli*. We thus suspected RIP113 to carry a *psiB* gene as R64*drd* and R100 plasmids. This was confirmed by PCR amplification with *psiB*-specific primers (data not shown). This finding is supported by another IncN plasmid which has been sequenced: the R64 plasmid carries a gene named *stbA* (locus R46_027), presenting 42% DNA sequence identity with *psiB*_F_.

**Figure 2:**
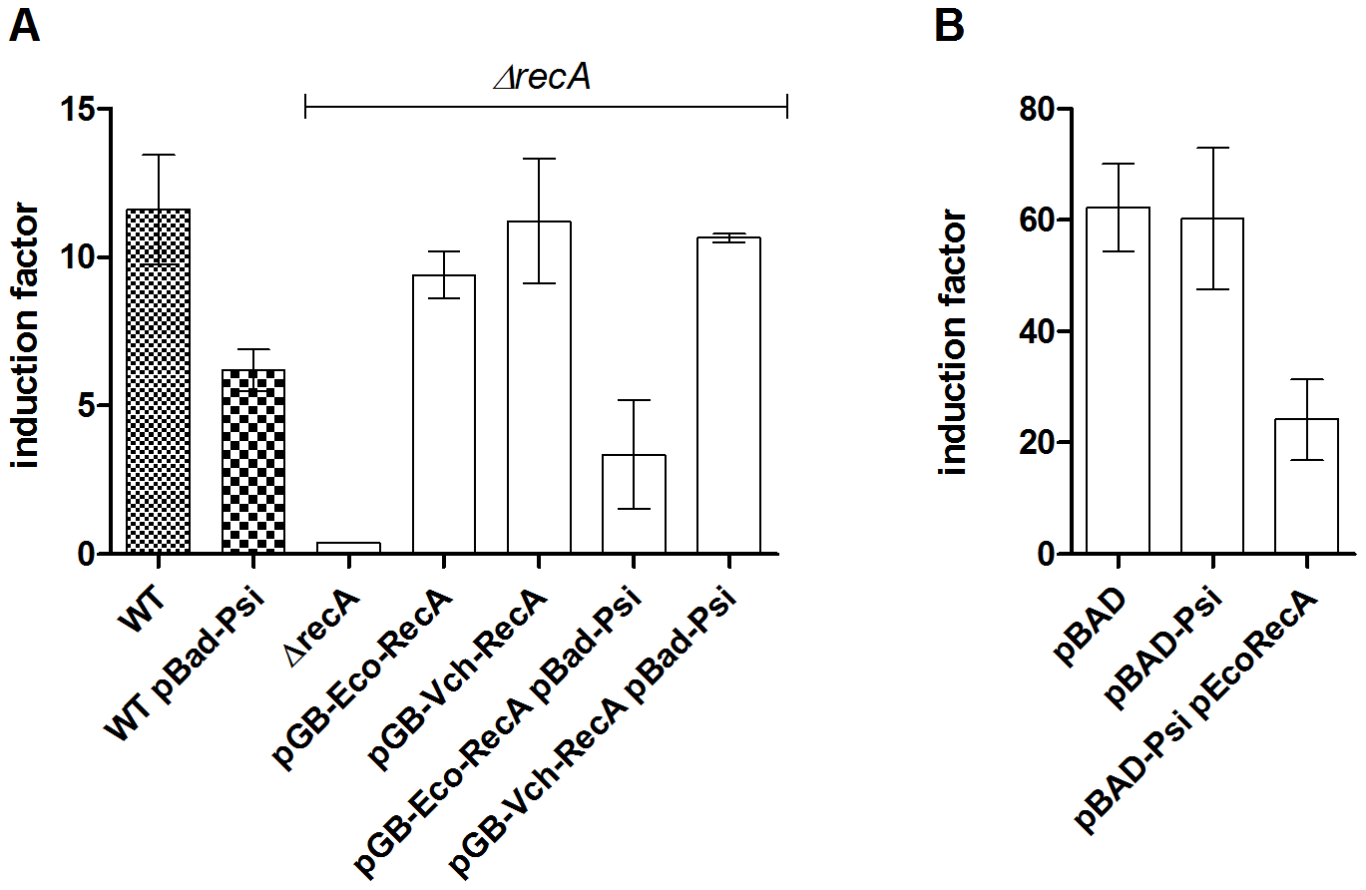
PsiB alleviates SOS induction in *E. coli* but not in *V. cholerae* because of impaired interaction with RecA*_Vch_*. β-gal tests showing SOS induction following MMC treatment. A: *E. coli* MG1655 *sfiA::lacZ*. B: *V. cholerae recN::lacZ*. Overnight cultures of *E. coli* 7651 and *V. cholerae* 7453 were diluted 100× in LB containing 0.2% arabinose and grown until OD∼0.5. SOS was induced for 1h with 0.2µg/ml MMC and β-gal tests were performed as described [61]. No MMC was added to control cultures.

We observed a strong induction of SOS by the same 3 plasmids in *V. cholerae* (Figure 1C), suggesting that the *psiB* gene is either not expressed in *V. cholerae* or that its product is not active in this species (R64*drd* and R100-1 do not replicate in *V. cholerae*, thus no activity per conjugant could be calculated). Moreover, SOS induction is continuously high for R64*drd* and R100-1 plasmids after ∼60 min, whereas SOS induction declined after 60 min for RP4, R6K*drd* and R388, as mentioned above. We were unable to delete *psiB* from R64*drd* and thus could not check if in its absence SOS induction would be restored in *E. coli*. The reason for the unsuccessful cloning attempts could be the presence of several genes (such as *ssb* coding the single strand binding protein, anti-restriction gene *ardA*, or *flm*/*hok*) in the same region where ORFs and regulatory regions overlap [9], [11], [39]–[41], such that deletion of *psiB* could have unpredicted consequences on plasmid transfer and replication. Instead, *psiB* from R64*drd* was cloned and over-expressed from a pBAD plasmid, under the control of the arabinose inducible promoter. SOS induction after mitomycin C (MMC) treatment was measured in *E. coli sfiA::lacZ* and *V. cholerae recN::lacZ* containing either empty pBAD or pBAD-PsiB+ plasmids. As previously published [31], MMC treatment induced SOS in *E. coli* and *V. cholerae* (Figure 2). SOS induction was strongly reduced in *E. coli* when PsiB was expressed from pBAD (6 fold induction instead of 11.6 fold, Figure 2A) whereas SOS induction was insensitive to PsiB expression in *V. cholerae* (∼60 fold induction with and without PsiB over-expression, Figure 2B). These results show that the *psiB*_R64*drd*_ (and presumably the *psiB*_R100-1_ which presents 85% identity to *psiB*_R64_) is expressed during conjugation in *E. coli* and inhibits the SOS response, whereas in *V. cholerae*, *psiB*_R64*drd*/R100-1_ has no or very little anti-SOS activity, allowing R64*drd* and R100-1 transfer to induce SOS. The fact that R64 and R100-1 are narrow host range enterobacterial plasmids [40], [42] and do not replicate in *V. cholerae*, can explain the continuous induction we observe. Entering plasmid DNA is not replicated and new rounds of conjugation can carry on, resulting in continuous re-induction of SOS.

### PsiB from narrow host range plasmids functions in a species-specific manner

The *psiB* anti-SOS function is found in several narrow host range plasmids belonging to the IncFI, IncFII, IncI1, IncK and IncN incompatibility groups [9] (and results obtained by blasting PsiB on GenBank plasmid sequences). These plasmids replicate in bacteria from the genera *Enterobacter*, *Escherichia*, *Salmonella* and *Klebsiella*. Bagdasarian and colleagues have suggested that PsiB could interact with RecA to inhibit its ability to induce SOS [16]. It was recently shown that PsiB binds to RecA in solution [43]. PsiB would then inhibit SOS by preventing RecA nucleofilament formation on ssDNA. Since PsiB from R64, R100-1 and RIP113 plasmids does not inhibit the SOS response in *V. cholerae* when over-expressed, we hypothesized that PsiB would be deficient in interacting with RecA_Vch_. Our β-gal tests show that when expressed in *V. cholerae* together with RecA_Eco_, PsiB reduces the SOS response from 60 fold to 24 fold induction (Figure 2B). Consistently, when co-expressed with RecA_Vch_ in an *E. coli* Δ*recA* strain, PsiB does not alleviate SOS (Figure 2A, note that RecA_Vch_ is active in *E. coli*). Finally, expression of RecA_Eco_ in the *E. coli* Δ*recA* strain complements SOS induction alleviation by PsiB. Altogether, these data suggest that PsiB is functional only in bacterial species where its carrier plasmids normally reside (here *E. coli*), thus antagonising RecA in a species-specific manner.

On the other hand, we showed that the RIP113 plasmid isolated from Salmonella, an enterobacterium, also carries the *psiB* gene and behaves like R64*drd* and R100-1 in inhibiting SOS in *E. coli* but not in *V. cholerae*. Unlike these two plasmids, RIP113 replicates in *V. cholerae* but since it was isolated in *Salmonella*, and to our knowledge IncN plasmids have not been described in *V. cholerae* so far, we considered that *V. cholerae* is not one of its usual hosts. To our knowledge PsiB is present only in narrow host range plasmids. We conclude that PsiB functions in a species-specific manner.

### Conjugation induces the integron integrase

**Figure 3:**
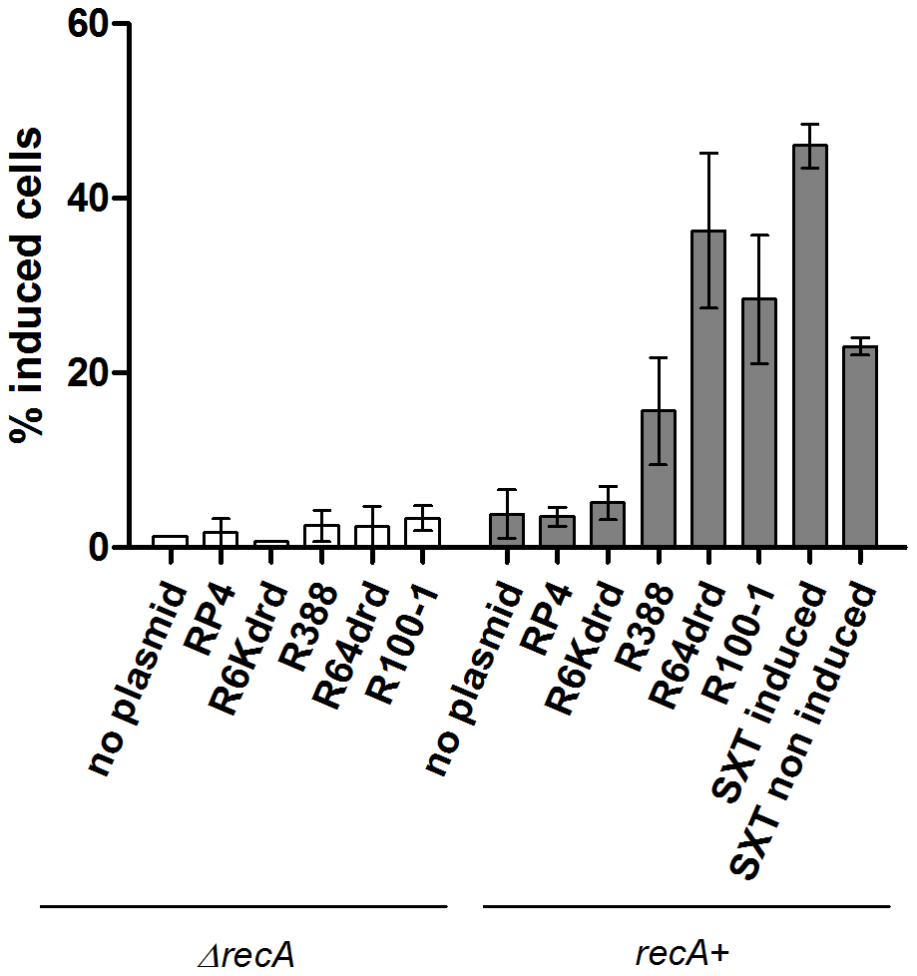
Conjugation induces *V. cholerae* integron integrase *intIA* expression. Donor and recipient (7093::p4640 *recA*+ and *ΔrecA*) strains were grown until OD 0.2, mixed at a 1∶1 ratio and incubated overnight on filter. % of GFP-induced cells was measured by flow cytometry.

It was recently shown that the integron integrase is regulated by the SOS response [31]. We showed above that conjugational DNA transfer induces SOS. We then addressed whether conjugation affects *V. cholerae* IntIA SI integrase expression levels. To do this, we constructed a *V. cholerae* reporter strain containing a translational fusion between *intIA* and *gfp* (7093::p4640), and used flow cytometry to determine the fraction of cells where the integrase-GFP fusion was induced. As expected, no induction was observed in the *ΔrecA* control strain (Figure 3). In the *recA*+ strain we observe no induction after conjugation with RP4 and R6K*drd* (Figure 3). Alternatively, the integrase expression increased 2.8 fold when the strain is conjugated with R388, and 5.3 and 6.2 fold with R64*drd* and R100-1, respectively. In β-gal SOS induction tests shown earlier, RP4 and R6K*drd* also yielded a lower induction in total population graphs (Figure 1B). Note that β-gal induction reflects the *recN* promoter, which is more strongly expressed than the *intI* promoter. Our results imply that the SOS induction during RP4/R6K*drd* conjugation may not reach sufficiently high levels to induce the integrase reporter used in flow cytometry experiments.

Finally we tested mating of *E. coli* carrying an SXT element with *V. cholerae*. SXT transfer is induced through induction of SOS when the donor is treated with MMC [37]. Transfer of the SXT element into *V. cholerae* increased *intIA* promoter activity 12 fold compared to a plasmidless control and was 2 fold higher than uninduced cells (*i.e.* without MMC treatment of donor).

### Conjugation triggers IntI1 integrase-dependent recombination

**Figure 4:**
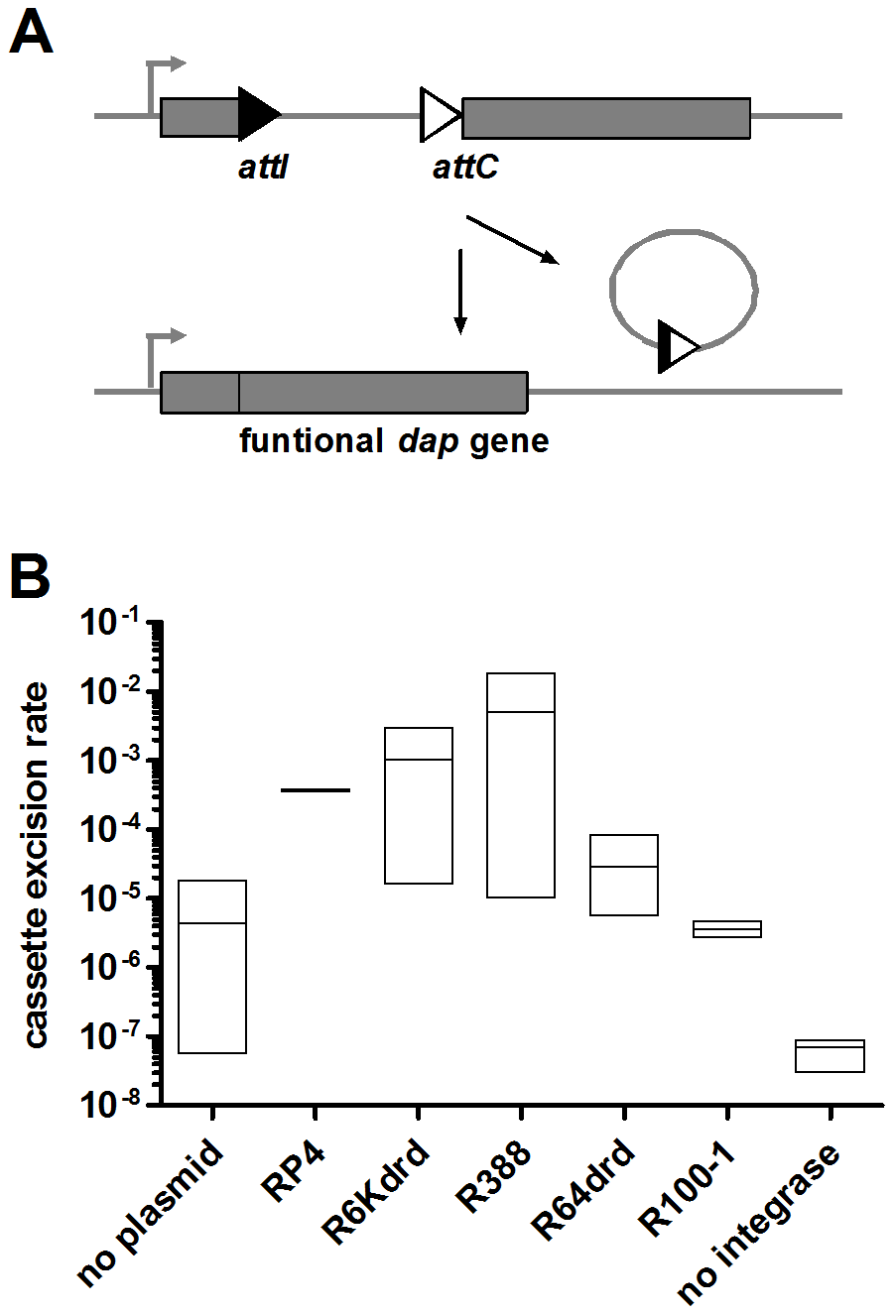
Conjugation increases IntI1-dependent cassette excision rate in E. *coli*. A: experimental setup. 7949 strain contains plasmid p7755 carrying *intI1* under the control of its natural LexA-regulated promoter. B: cassette excision rate was calculated by counting recombined *cfu* (Dap+) over total *cfu*. “No plasmid” means that recipient 7949 p7755 was mixed with empty donor. “No integrase” means that recipient 7949 without p7755 was conjugated with donor containing a conjugative plasmid.

We have shown that conjugation induces SOS in the recipient bacteria and flow cytometry analysis clearly shows that the integron integrase is induced during conjugation in *V. cholerae*. In a first set of experiments, we wanted to test if the SOS induction leads to a higher activity of the integrase promoter in *E. coli*, using the class 1 integrase IntI1. We developed an experimental strategy in an *E. coli* strain that contains an insertion in the *dapA* gene (7949). This strain is unable to synthesize DAP (2,6-diaminopimelic acid), and as a result is not viable without DAP supplemented in the medium. The insertion in *dapA* is flanked by two specific recombination sites, *attI* and *attC*. Integrase expression causes site-specific recombination and excision of the synthetic cassette, restoring a functional *dapA* gene and allowing the strain to grow on DAP-free medium (Figure 4A). We transformed in this *dapA*- strain a multi-copy plasmid (p7755) carrying the *intI1* gene under the control of its natural SOS regulated promoter. The recombination rate due to integrase expression is calculated as the ratio of the number of cells growing in the absence of DAP over the total number of cells. Figure 4B shows the cassette excision rate in *E. coli* 7949 p7755 after conjugation with different conjugative plasmids. In the absence of a conjugative plasmid in the donor cell, the spontaneous excision rate is about 10^−5^, which reflects the stringency of the *intI* promoter. Conjugation with R6K*drd* and R388 increases excision rate to 10^−3^ and 10^−2^ respectively, whereas conjugation with R64*drd* does not increase significantly beyond the basal recombination level. RP4 yields an intermediate level of DAP+ cells, which is compatible with its intermediate SOS induction level in *E. coli*. These results are consistent with SOS induction results in *E. coli*, and as expected, there is a correlation between SOS induction and integrase induced cassette recombination. To confirm that cassette recombination is due to integrase expression, we performed the same experiment in strain 7949 lacking the integrase carrying plasmid p7755, and no cassette excision was observed (<10^−8^). We conclude that conjugation with *psiB* deficient plasmids in *E. coli* induces the expression of the integrase from the *intI1* promoter, and thus triggers cassette recombination.

### Conjugation triggers IntIA–mediated cassette recombination in the *V. cholerae* superintegron

**Figure 5:**
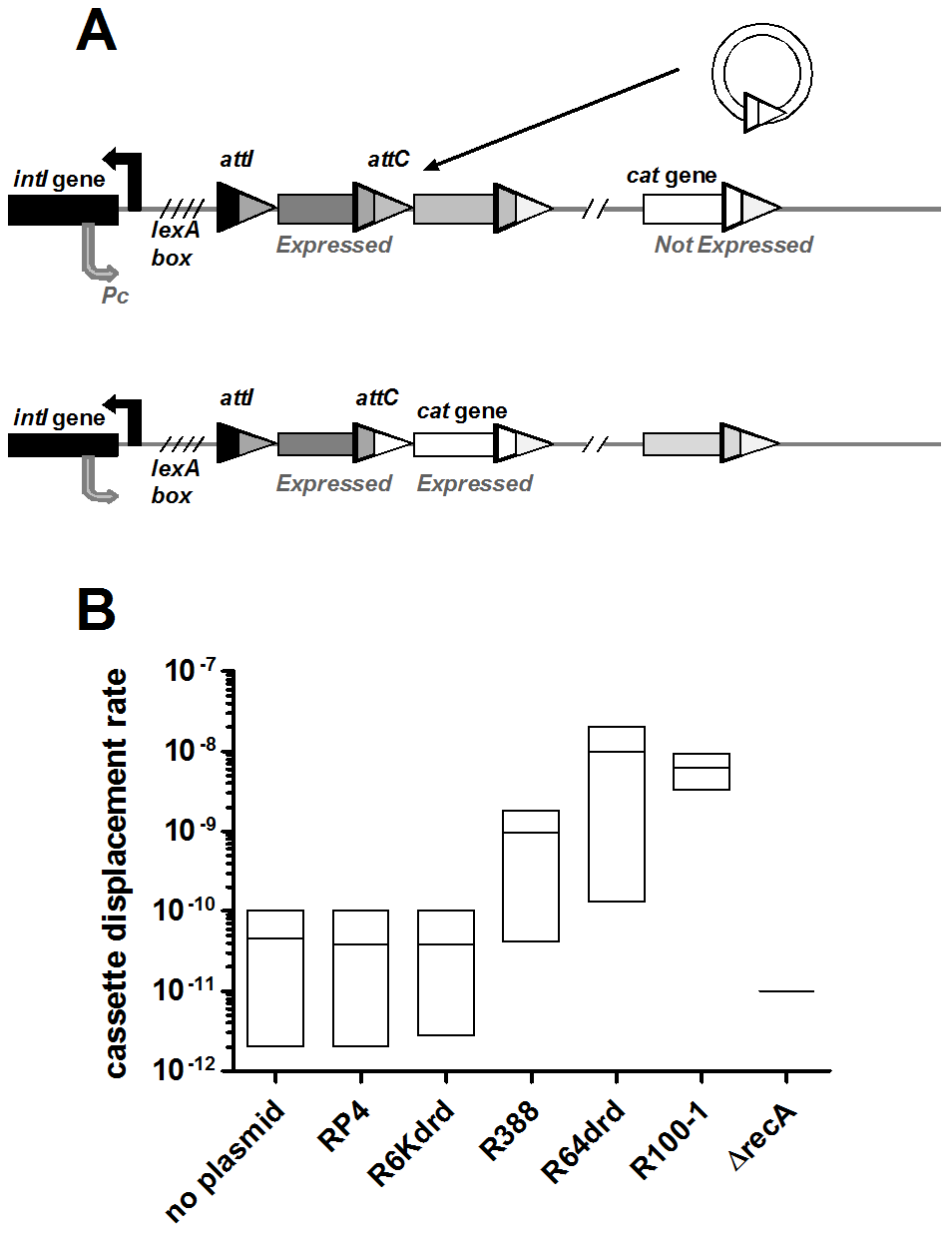
Conjugation triggers IntIA-dependent cassette recombination in *V.cholerae* superintegron. A: model: the *catB* cassette moved to 2^nd^ position on the SI. B: cassette displacement rate, *i.e* Cm-R *cfu* over total *cfu*. “No plasmid” means that recipient was mixed with empty donor.

In the cassette excision experiment described above, we used a multicopy plasmid expressing the *intI1* integrase in *E. coli*. Since conjugation induces the SOS response and in turn expression of the integron integrase in *V. cholerae*, we addressed in a second set of experiments whether conjugation in wild type *V. cholerae* can trigger recombination events in the superintegron. The *V. cholerae* SI carries a promoterless *catB* cassette that is not expressed in *V. cholerae* laboratory strain N16961 because it is located 7 cassettes (approximately 5000 bp) downstream of the Pc promoter [44]. When expressed, the *catB* gene confers resistance to chloramphenicol (Cm). We tested if conjugation can spontaneously yield Cm-resistant (Cm-R) *V. cholerae* cells, *i.e.* if IntIA is induced and recombines the *catB* cassette to a location allowing its expression (Figure 5A).

Our results show that when the donor strain does not carry any conjugative plasmid, the rate of CmR cells is about 7.10^−11^ (Figure 5B). Consistent with the *intIA* induction results, conjugation with RP4 and R6K*drd* did not increase this frequency (6.10^−11^). Conjugation with R388, R64*drd* and R100-1 increased the CmR *cfu* appearance rate 28 fold, 280 fold and 140 fold, respectively. To verify that this increase was dependent on SOS, we deleted *recA* in the recipient strain and found that the conjugative plasmids yielded a rate of CmR lower than 10^−11^ for all plasmids (no colony observed).

To determine if these events corresponded to IntIA mediated cassette rearrangement, we performed a PCR analysis with primers in the Pc promoter and at the beginning of the *catB* cassette. In the wild type strain, this PCR amplifies a band of about 5000 bp. In the CmR colonies, the PCR amplified a band of 1432 bp (Figure S2A). Sequencing confirmed that the *catB* cassette had been relocated closer to the Pc promoter. *catB* was now present in second position, after the first cassette, compatible with an excision and integration in the first *attC* site downstream of *attI*. We know that IntIA can promote recombination between two *attC* sites [45]. The first cassette coding for a hypothetical protein downstream of *attI* may be important for viability under laboratory conditions. Alternatively, there may be a strong promoter in this first cassette, allowing a better expression of the *catB* cassette that could be insufficiently expressed from any other location under our selective conditions (involving high Cm concentrations).

In order to determine if cassettes between the Pc promoter and *catB* gene were deleted after rearrangement – i.e. if *catB* moved because cassettes were deleted or because it was re-integrated – we performed PCR analysis with several oligonucleotides amplifying cassettes located between *attI* and *catB* in *V. cholerae* N16961. We found that these cassettes were still present in the genome of the Cm-resistant clones (data not shown), showing that they were not deleted, and indicating that *catB* was relocated by recombination events.

To further address if other cassette rearrangements had occurred in those cells after conjugation, we isolated the genomic DNA (gDNA) from three CmR colonies obtained after conjugation. We digested gDNA with AccI, which has 35 restriction sites evenly distributed within the SI. We then hybridized with a mix of 10 probes complementary to 17 cassettes in the SI. The southern blot (Figure S2B) clearly shows that 2 of the 3 colonies have different hybridization profiles compared to the original N16961 gDNA, confirming that several cassettes (other than *catB*) have moved within the SI after conjugation induced SOS.

We conclude that conjugation with strong SOS inducing plasmids, R388, R64*drd* and R100-1, increases IntIA expression levels and promotes cassette rearrangements at a 100 fold to 1000 fold higher rate than under non stressful conditions. Although conjugation of R6K*drd* strongly induced the SOS response, it did not have any effect on *intIA* expression and cassette recombination in our experimental setup. Even though it is possible that R6K*drd* encodes an uncharacterized function able to specifically prevent the IntI expression during the SOS induction, we think this observation is most likely due to an insufficient sensitivity of our setup.

## Discussion

We showed that conjugation of RP4, R6K*drd*, R388 and Rs-a plasmids, that do not carry any anti-SOS function, induces the SOS response in recipient *E. coli* and *V. cholerae* cells. Alternatively, plasmids R64*drd*, R100-1 and RIP113 that do carry the anti-SOS *psiB* gene do not induce SOS when the recipient cell is *E. coli*, while the SOS response is induced in *V. cholerae*. Finally, the SXT element (here integrated in the *E. coli* chromosome) is also able to induce SOS when it transfers to recipient *V.cholerae*.

### Induction of SOS during conjugation

It has been shown that during inter-species Hfr conjugation, SOS is induced in the host cell [46], [47]. It was proposed that the low level of homology prevents rapid recombination of incoming DNA into the chromosome and thus dramatically enhances the SOS induction. This suggests that SOS induction levels may reflect the ability of RecA to find homologous DNA and initiate strand exchange [48]. In the case of plasmid conjugation, there is no homology with the bacterial chromosome, explaining the very high SOS induction levels we observe. Moreover, SOS induction proceeds as a wave. At the early stages of SOS induction, the concentration of LexA decreases as it is self-cleaved, then LexA synthesis is induced at later stages, and if ssDNA does not persist, the SOS induction level gradually decreases. This is what happens when RP4, R388 and R6K*drd* conjugate into *E. coli* cells (and RP4 in *V.cholerae*), and explains the bell-shaped induction curves we obtained. After 1h, nearly all the recipient cells are conjugants and no new conjugation is initiated because of plasmidic entry exclusion systems [49]. As conjugation or establishment rates are very low for R388 and R6K*drd* in *V. cholerae*, plasmidless host cells are always present in the total population so we observe a plateau reflecting new rounds of conjugation during the course of the experiment. The fact that R64*drd* and R100-1 are the strongest inducers in *V. cholerae* could thus be due to the inability of these plasmids to synthesize the complementary DNA strand and establish themselves in *V. cholerae*, increasing the prevalence of ssDNA accessible to RecA binding. Moreover, this strongly suggests that abortive conjugation induces SOS, which explains the fact that we are able to detect SOS induction in the total population despite the lack of transconjugants in *V. cholerae*. This is consistent with data showing very high induction values when we calculate the SOS induction in conjugants only. The plateau of induction in the whole population points to a “permanent conjugation state” where ssDNA enters the host cell, induces SOS, but does not replicated, and a new round of conjugation begins.

Another interesting observation is that SOS induction does not seem to prevent conjugation. Indeed, the conjugation rate for all plasmids (in *E. coli* for instance) is approximately the same after 2h of mating even though they induce SOS differently at the beginning of mating. To test this point, we used recipient cells already induced for SOS by pre-incubation with MMC, and these cells yielded the same conjugation rates in *E. coli* for a given plasmid regardless of the SOS induction level (Figure S1).

### Narrow host range plasmids inhibit the SOS response in their natural host

SOS induction is due to RecA binding to ssDNA. We have shown above that plasmids R64*drd*, R100-1 and RIP113 that carry the anti-SOS *psiB* gene do not induce SOS when the recipient cell is *E. coli*. PsiB has been shown to interact with RecA*_Eco_ in vitro* [43] and *in vivo* (Figure 2), preventing it from binding to ssDNA and inducing the SOS response. Even though RecA_Vch_ and RecA_Eco_ show 79% protein identity, our data suggests that PsiB is impaired in its interaction with RecA*_Vch_ in vivo* (Figure 2), explaining why PsiB does not strongly reduce the SOS induction in *V. cholerae* as it does in *E.coli*. The presence of the *psiB* anti-SOS function in narrow host range plasmids such as R64 and R100 suggests that the dissemination strategies of narrow and broad host range plasmids could be distinct. Induction of the SOS response can be potentially detrimental to the host cell because of the induction of mutagenic polymerases or cell division arrest (like *E.coli sfiA*) [50]. Thus, it is tempting to speculate that narrow host range plasmids use their anti-SOS gene as a furtive strategy to hide from their customary host and thus prevent the host cell from being stressed and change its own or the incoming plasmid DNA. Note that by narrow host range plasmids, we mean plasmids that only replicate in a restricted number of bacteria (such as R64) but also plasmids that are found only in a few kinds of hosts in nature, even though they are able to replicate in others, such as the RIP113 originally in *Salmonella*.

### SOS induction during conjugation leads to chromosome rearrangements

One consequence of SOS induction during conjugative DNA transfer is the triggering of integron cassette recombination. Conjugation with strong SOS inducer plasmids R388 and R6K*drd* in *E. coli* increases expression of IntI1 from its SOS regulated natural promoter leading to an increased RecA-dependent cassette excision rate, whereas plasmids R64*drd* and R100-1 that do not induce SOS in *E. coli* do not trigger cassette recombination in our *E. coli* cassette recombination assay. These results highlight the existence of a link between conjugation and site-specific recombination, leading to genome evolution. We also showed that conjugation triggers cassette recombination in the natural context of the SI carried in wild type *V. cholerae*. Plasmids R388, R64*drd* and R100-1 strongly induce SOS (and *intIA*) in *V. cholerae* and significantly increases the cassette recombination rate.

Our results highlight the link between conjugative HGT and genome evolvability in *V. cholerae*. Since conjugation induces integrase activity, one can consider conjugative plasmids as both vehicles for cassette dissemination and cassette shuffling for those already present in the SI. Indeed, some plasmids such as R388 [51] and R64 [52] carry an RI platform that can acquire new cassettes and transmit them to a new host by conjugation. It was shown that R388 can incorporate the *catB* cassette from the *V. cholerae* SI and transfer it to other bacteria [44]. Here we observed the displacement of a *cat* cassette catalyzed by the *V. cholerae* IntIA in its natural context. Conjugation can thus bring new cassettes but also favour their integration into the host chromosomal integron by inducing SOS.

### Conjugation, SOS, and genome evolution

Cassette recombination upon conjugation could be a widespread mechanism, since conjugation is a naturally occurring phenomenon in highly concentrated bacterial environments, such as the host intestinal tract (see for example [17]–[19], [21]), biofilms [53]–[55] forming in the aquatic environment where *V. cholerae* grows, or even on medical equipment in hospitals [56]. Moreover, no mutation has been found in the bacterial chromosome that can prevent the uptake of conjugative DNA, meaning that bacteria cannot avoid being used as recipient cells [57]. By inducing the SOS response, incoming DNA triggers its own recombination not only through integrase induction but also homologous recombination, promoting genomic rearrangements.

Another important effect of SOS induction is the derepression of genes implicated in the transfer of integrating conjugative elements (ICEs), such as SXT from *V.cholerae*, which is a ∼100 kb ICE that transfers and integrates into the recipient bacterial genome, conferring resistance to several antibiotics [37]. Moreover, different ICEs are able to combine and create their own diversity in a RecA-dependent manner via homologous recombination [35], [36], and also, as observed here for SXT transfer in *V. cholerae*, by inducing SOS following transfer. Thus, SOS induction leads to genetic diversification of these mobile elements and to their transfer to surrounding bacteria, spreading antibiotic resistance genes, among others.

Conjugation induced SOS is thus one of the mechanisms allowing bacteria to evolve in their natural niches, creating the diversity that allows them to adapt to new environments and survive. Under conditions where SOS is prevented (by bacterial means such as the PsiB system or exogenously), cassette recombination is decreased to experimentally undetectable levels, showing that SOS induction plays an important role in adaptation, and can be used by broad host range plasmids to adapt to a new host. Consistently, narrow host range plasmids that do not need to adapt to a new host, express an SOS inhibitor to maintain the integrity of the plasmid DNA and host genome. This connection between host range and SOS induction needs to be expanded to a larger range of plasmids to determine its general character. A significant association between laterally transferred genes and gene rearrangements was already suggested in [58], which is consistent with our data, when we consider that SOS induction plays a major role in gene rearrangements. Remarkably, induction of SOS considerably enhances genome duplications and mutagenesis [59]. Further work is needed to test whether other HGT mechanisms, such as transformation, also induces SOS; considering that many bacterial species, like *V. cholerae* for instance, are naturally competent [60]. It would be interesting to investigate if SOS is induced in the gut of the host animal. If this were the case, inhibiting the bacterial SOS response would become an ideal target to prevent the acquisition of antibiotic resistance genes, and could be used in combination with antibiotics for the treatment of infections.

## Materials and Methods

For strain and plasmid constructions, and oligonucleotide list, see Text S1 and Tables S1 and S2.

### Conjugation and β-galactosidase tests

Overnight cultures of donor and recipient cells were diluted 100× in LB and grown until OD∼0.5. Donor and recipient cells were then mixed in 1∶1 ratio on 0.045µm conjugation filters on LB plates preheated at 37°C. At each time point, a filter was resuspended in 5ml LB and dilutions were plated on selective plates to count (i) conjugants and (ii) total number of recipients. For details, see Text S1.

β-gal tests were performed on these cultures as described ([61] and Text S1). According to the Miller formula:
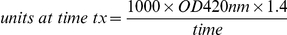
and we calculated:
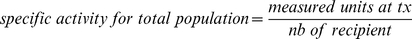
and
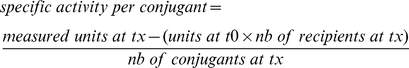
where 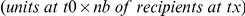 is the basal expression per cell when SOS is not induced. For details, see Text S1.

SOS induction tests using MMC were performed as published [31].

### Flow cytometry

The same conjugation assay was performed overnight for the flow cytometry experiments. For each experiment, 100000 events were counted on the FACS-Calibur device. For details, see Text S1 and Figure S3.

### Cassette excision measurements

Described in the Results section. For details, see Text S1.

### Cassette displacement measurements

The recipient strain was *V. cholerae* N16961 (Cm sensitive 5µg/ml Cm). The donor strain was DH5α or a *dap-* derivative (Π1) for counter selection of Cm-R plasmids. Conjugations were performed as described for 4h. Filters were resuspended, centrifuged and the pellet was plated on LB medium containing 25µg/ml Cm. PCR screenings were performed using oligonucleotides cat2/i4 and the GoTaq polymerase. Oligonucleotides 896 to 905 were used to verify the presence of other cassettes.

## Supporting Information

Figure S1 SOS induction does not affect conjugation rate. Recipient *E. coli* and *V. cholerae* were grown in LB containing 0.2 µg/ml MMC up to OD ∼0.5. Conjugations were performed for 1h as described in the Materials and Methods.
(0.41 MB TIF)

Figure S2 Cassette displacement after conjugation within the *V. cholerae* SI. A: Displacement of *catB* cassette within the SI after R64/R388 conjugation. Oligonucleotides used for PCR reactions were i4/cat2. B: Southern blot visualization of the *V. cholerae* SI cassette array reorganization after SOS induction. gDNA from *V. cholerae* N16961 and CmR derivatives were digested by AccI and probed with a mix of oligonucleotides corresponding to different cassettes. Oligonucleotides anneal to cassettes VCA0291 to VCA0295, VCA0298 to 0300, VCA0329, VCA0343, VCA0354 to VCA0356, VCA0361, VCA0364 to VCA0366 and are listed in Table S2.
(0.13 MB TIF)

Figure S3 Example of analysis of flow cytometry data. Y axis: cell count, X axis: fluorescence. Red curve is obtained by counting V. cholerae cells with constitutive GFP expression. Black curve represents a mating mixture of plasmid free *E. coli* and *V. cholerae* carrying the intI-gfp fusion. This mixture was taken as negative reference. Cells showing a fluorescence in the M1 region (intersection point of black and red curves and further right) are considered as induced cells. Green curve is an example of the data obtained: it represents a mixture of *E. coli* carrying plasmid R100-1 and *V. cholerae*.
(0.05 MB TIF)

Table S1 Bacterial strains and plasmids.
(0.09 MB DOC)

Table S2 Oligonucleotides used in this study.
(0.05 MB DOC)

Text S1 Supplementary experimental procedures.
(0.07 MB DOC)
