# Supplementary figures and images for: Conjugative DNA Transfer Induces the Bacterial SOS Response and Promotes Antibiotic Resistance Development through Integron Activation
Source: PLoS Genet. 2010 Oct 21;6(10):e1001165. doi: 10.1371/journal.pgen.1001165 (PMC2958807; doi:10.1371/journal.pgen.1001165)

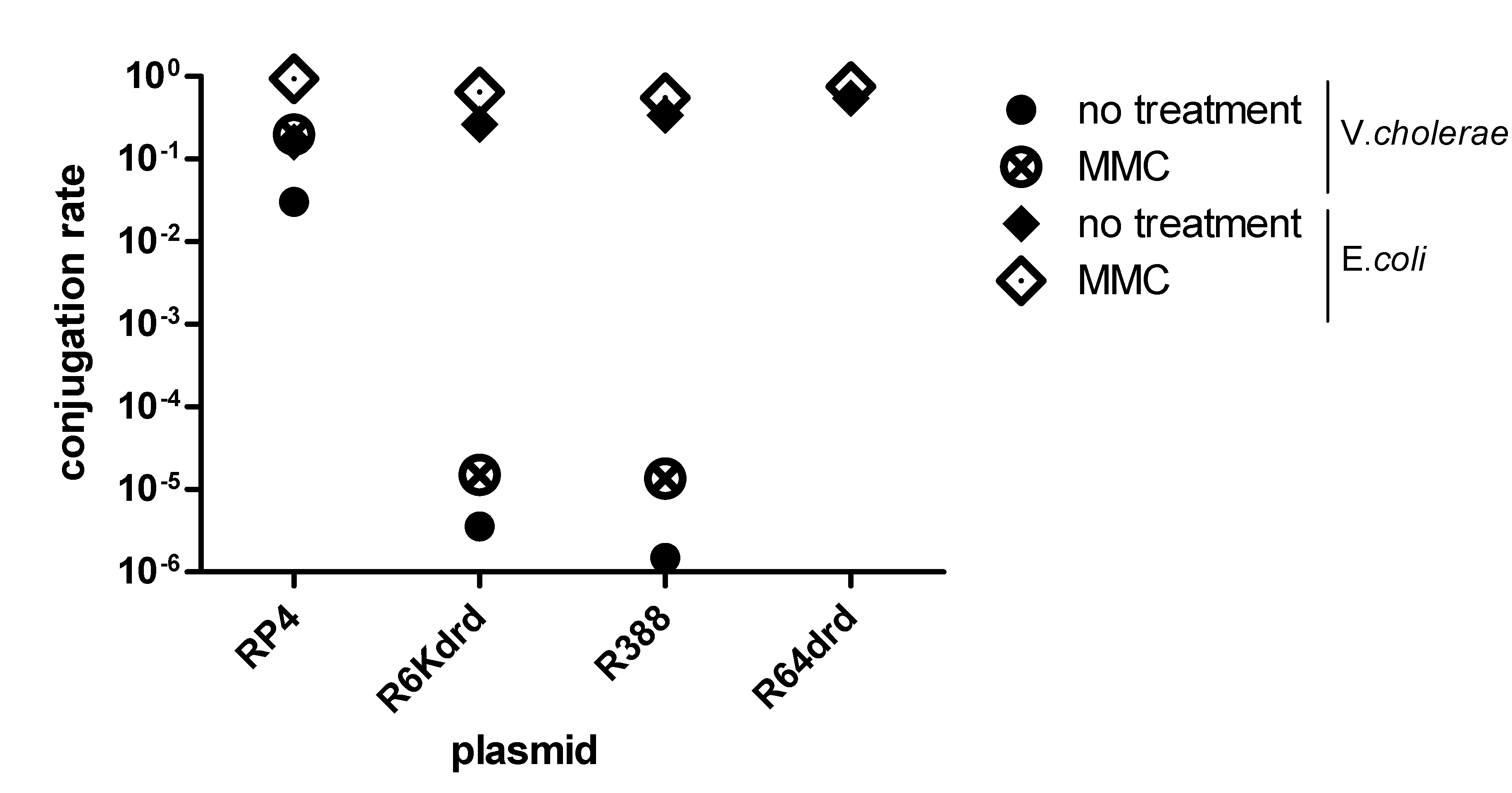

Supplement: Figure S1 — SOS induction does not affect conjugation rate. Recipient E. coli and V. cholerae were grown in LB containing 0.2 µg/ml MMC up to OD ∼0.5. Conjugations were performed for 1h as described in the Materials and Methods. (0.41 MB TIF) [file pgen.1001165.s001.tif]

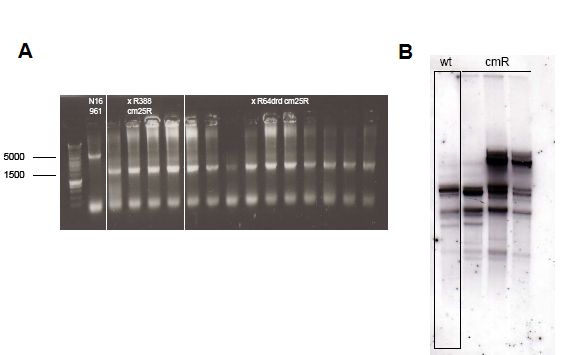

Supplement: Figure S2 — Cassette displacement after conjugation within the V. cholerae SI. A: Displacement of catB cassette within the SI after R64/R388 conjugation. Oligonucleotides used for PCR reactions were i4/cat2. B: Southern blot visualization of the V. cholerae SI cassette array reorganization after SOS induction. gDNA from V. cholerae N16961 and CmR derivatives were digested by AccI and probed with a mix of oligonucleotides corresponding to different cassettes. Oligonucleotides anneal to cassettes VCA0291 to VCA0295, VCA0298 to 0300, VCA0329, VCA0343, VCA0354 to VCA0356, VCA0361, VCA0364 to VCA0366 and are listed in Table S2. (0.13 MB TIF) [file pgen.1001165.s002.tif]

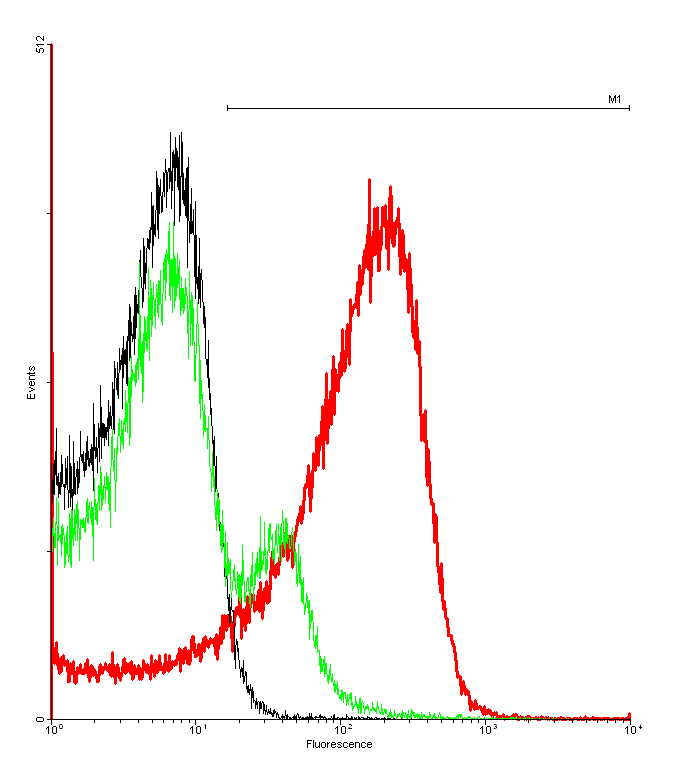

Supplement: Figure S3 — Example of analysis of flow cytometry data. Y axis: cell count, X axis: fluorescence. Red curve is obtained by counting V. cholerae cells with constitutive GFP expression. Black curve represents a mating mixture of plasmid free E. coli and V. cholerae carrying the intI-gfp fusion. This mixture was taken as negative reference. Cells showing a fluorescence in the M1 region (intersection point of black and red curves and further right) are considered as induced cells. Green curve is an example of the data obtained: it represents a mixture of E. coli carrying plasmid R100-1 and V. cholerae. (0.05 MB TIF) [file pgen.1001165.s003.tif]
