# Supplementary material for: Conjugative DNA Transfer Induces the Bacterial SOS Response and Promotes Antibiotic Resistance Development through Integron Activation
Source: PLoS Genet. 2010 Oct 21;6(10):e1001165. doi: 10.1371/journal.pgen.1001165 (PMC2958807; doi:10.1371/journal.pgen.1001165)
Supplement: Table S1 — Bacterial strains and plasmids. (0.09 MB DOC) [file pgen.1001165.s004.doc]

| **Strain Genotype** | **Strain number** | **Phenotype of interest** | **AB-Res** | **Construction or reference** |
| --- | --- | --- | --- | --- |
| ***Escherichia coli*** |  |  |  |  |
| *sfiA::lacZ* | 7651 | *lacZ* under control of SOS |  | B. Michel |
| *recA::tn10* | 6670 | tc -resistance cassette inserted in *recA* | tc | B. Michel |
| *sfiA::lacZ recA::tn10* | 7713 | *lacZ* under control of SOS; SOS defective strain | tc | This study |
| *hsdR recA* | DH5α | *Defective for SOS; Used as donor for R-plasmid conjugations* |  | Laboratory collection |
| *dap*-N-ter::[attI-cassette-attC]::dap-C-ter | 7949 | defective for DAP, becomes DAP+ after site specific recombination | km | This study |
| DH5α *Δ*thy::*Pir+* | Π1 | needs thymine in medium | erm | (Demarre et al., 2005) |
| MG1655:: *ΔdapA*::(*erm,pir* ) RP4-2-Tc::Mu | β2163 | needs DAP in medium | km | (Demarre et al., 2005) |
| β2163 GyrA462- tc | β3914 | *ccdB-Resistant*, needs DAP in medium  used to deliver pSW7848-derived deletion vectors | tc | (Val et al., 2008) |
| *recA:: tc dapA::erm-pir* | 403 | Needs DAP in medium to survive; Used as suicide donor for R-plasmid conjugations | tc, erm | w40, Laboratory collection |
| CAG18439SXT | 8266 | SXT integrated in the *E.coli* chromosome | tc | (Burrus and Waldor, 2003) |
| ***Vibrio cholerae*** |  |  |  |  |
| N16961 |  | *Vibrio cholerae* El Tor strain |  | Laboratory collection |
| N16961 *recN-lacZ* | 7453 | *lacZ* under control of SOS | km, cm | (Guerin et al., 2009) |
| N16961 *ΔrecA:: km* | 8218 | Wild type strain (used for *catB* cassette displacement assays);Defective for SOS induction | km | Deletion of *recA* from strain N16961 with plasmid pMEV97 |
| *V.cholerae* A1552 *Δ*dns | 7093 | *Vibrio cholerae* El Tor transformable strain; RifR | rif | M. Blokesch |
| A1552 *Δ*dns *ΔrecA::km* | 7955 | Defective for SOS induction | rif, km | Deletion of *recA* from strain 7093 with plasmid pMEV97 |
| **Plasmids** |  |  |  |  |
| pBAD-PsiB+ | 8147 | PsiB inducible by arabinose | ap | This study |
| pGB-RecA*_Vch_* | 8149 | *V.cholerae* *recA* expressed from own promoter (inducible by MMC) | sp | This study |
| pGB-ara-RecA*_Eco_* | 8152 | *E.coli* *recA* inducible by 0,2% arabinose | sp | B. Michel |
| pBAD-[*intI1lexAbox*]-*intI1* | 7755 | IntI1 expression regulated by LexA | ap | This study |
| pSW23T | 970 | *oriT*_RP4_, *oriV*_R6K_ ; Pir dependent replication | cm, km | (Demarre et al., 2005) |
| pSW7848 | 7848 | A ccdB containing derivative of pSW4426T ; R6K *ori*V , RP4 *oriT*, *ccdB-araC*; Pir dependent replication | Cm | (Le Roux et al., 2007; Val et al., 2008) |
| pMEV97 | 6993 | *Δ*recA:: km - Counter-selection *sacB* ; For recA deletion in *V.cholerae* | cm, km | (Val et al., 2008) |
| p4640 | 4640 | *gfp* in transcriptional fusion with *intIA* | cm, km | This study |
| pZE1-GFP | 8264 | Carries GFP | km | C. Beloin |
| pSU19-*oriT_F_* | 8267 | Carries the *oriT* of F plasmid | cm | A. Kaminski |

**Table S1: Bacterial strains and Plasmids**
