# Supplementary material for: Conjugative DNA Transfer Induces the Bacterial SOS Response and Promotes Antibiotic Resistance Development through Integron Activation
Source: PLoS Genet. 2010 Oct 21;6(10):e1001165. doi: 10.1371/journal.pgen.1001165 (PMC2958807; doi:10.1371/journal.pgen.1001165)
Supplement: Table S2 — Oligonucleotides used in this study. (0.05 MB DOC) [file pgen.1001165.s005.doc]

| **Number** | **Name** | **Sequence 5’-3’** |
| --- | --- | --- |
| **534** | psiB-1 | GCAGCAAGGGAGATACCG |
| **535** | psiB-a | CCTGTGAACGTACGCTC |
| **570** | Pint_in56_Fw | TTTTGAATTCGCGTAACGCGCTTGCTGCTTGGATGCCCGAGGCATAG  ACTGTACAAAAAAACAGTCATAACAAGCCATGAAAACCGCCAC |
| **272** | 3938_IntI_HindIII_Rw | ATGCAAGCTTGGATCCCCCCATAAAAAAACCCGCCGAAGCGGGTT  TTTACGTTATTTCTACCTCTCACTAGTGAGGGGCGGCAGCG |
| **299** | RecReport-F | TTTGAATTCGCGGCCGCTTCTAGAGTGAGCGCAACGCAATTAATG |
| **396** | dapA-attC-R | GCGACAATACTTCCCGTGAAGTAACGCTTGAATTAAGCCGCGCCGC |
| **399** | attC-dapA-DNheI-F | CAAGCGTTACTTCACGGGAAGTATTGTCGCGATTGTTACTCCGATGGATGAAAAAGGTAATGTCTGTCGGGCGAGCTTG |
| **353** | dapA-R | TTTGGATCCTTACAGCAAACCGGCATGCTTAAGC |
| **620** | int-d | CGAGAGCTCCTACCTCTCACTAGTGAGGGGCGGCAGCGCATCAAGCGGTG |
| **621** | int-f | TACGAGCTCATGAAAACCGCCACTGCGCCGTTACCACCGCTGCGTTCGGT |
| **735** | cat2 | CGCGGATCCGCGAACACTTAACAAAAACTGGG |
| **736** | i4 | AAACTGCAGTTGTTATGTGCGTGCTTTTGATC |
| **775** | ecopMP7-1 | GCGGTGCTCAACGGGAATCC |
| **776** | ecopMP7-a | AAAAATGCACCGGGGCCAGC |
| **896** | K7-1 | GTTGTTATGGTAGTATGCACCC |
| **897** | K7-a | AGCCAATTTTTACAGCTTAATATCAACC |
| **898** | K7-2 | GTGTTTAAGTTTGGTGTCATGC |
| **899** | K7-b | CCATTTTTTTTCGGTGGCGGCG |
| **900** | K7-3 | TTGCTCACACCTTAATGCGGCGTTAGG |
| **901** | K7-c | GGGGTGAGCAACGCAATACCG |
| **902** | K7-4 | CAGAGGCTGTTAGCTTGTTGAAAGCC |
| **903** | K7-d | CGATGCTTGTACTGGGTTGGGCAGTAGCC |
| **904** | K7-5 | ATGGCGTGGTCGTGTTTCGGGCCG |
| **905** | K7-e | TGTTGTGTTCTGTACTCATTTTGCC |
| **922** | vchrecA-1-eco | ATCGAATTCAAGCTTAGCATCTCGCAGCAGATCAGG |
| **923** | vchrecA-a-eco | GTAGAATTCGAGCTCTTGAGAGGCTGAACCTCTTTG |
| **1031** | cm-south-1 | CTGGGATTCCCTTAGATCAGC |
| **1032** | cm-south-a | CCGACCACTTCATAAGGTGCAACATCC |
| **1047** | o-intI4-bam-fw | AGTTCTTCTCCTTTGCTAGCCATATAGTTCTCACTGAATATTTAACTG |
| **1048** | o-intI4-rbs-gfp-rev | AGTTCTTCTCCTTTGCTAGCCATTATTAATACCCTCTAGATTGATTACTAAAGACGGGATAATGGGCTTA |
| **1049** | o-inti4-rbs-gfp-fw | TAAGCCCATTATCCCGTCTTTAGTAATCAATCTAGAGGGTATTAATAATGGCTAGCAAAGGAGAAGAACT |
| **1050** | o-gfp-pstI-rev | AACTGCAGTCATTATTTGTAGAGCTCATCCATGCC |

**Table S2 : Oligonucleotides used in this study**
