# Supplementary material for: Conjugative DNA Transfer Induces the Bacterial SOS Response and Promotes Antibiotic Resistance Development through Integron Activation
Source: PLoS Genet. 2010 Oct 21;6(10):e1001165. doi: 10.1371/journal.pgen.1001165 (PMC2958807; doi:10.1371/journal.pgen.1001165)
Supplement: Text S1 — Supplementary experimental procedures. (0.07 MB DOC) [file pgen.1001165.s006.doc]

**Supplemental Data**

**Construction of Plasmids and bacterial strains: p7755** was obtained by cloning the natural *intI1* (in56) promoter in front of *intI1* in p3938: *intI1* was amplified from p3938 (Guerin et al., 2009) with oligonucleotides 570 (containing the LexA box)/272 and cloned in EcoRI/HindIII sites in pBAD18.  **p8147 (pBad-PsiB+)** : *psiB* from R64*drd* was PCR amplified with oligonucleotides 534/535 and cloned in pTOPO plasmid using the invitrogen pTOPO-blunt kit. *psiB* was extracted from pTOPO-*psiB* by EcoRI digestion and cloned in the same restriction site in pBAD18. **p8149 (pGB-RecA*_Vch_*)**: *V.cholerae* *recA* was amplified from N16961 with its own promoter using oligonucleotides 922/923 and cloned in pTOPO-Blunt. pGB-ara-RecA*_Eco_* (p8152) was digested by EcoRI to remove *recA_Eco_* and vector pGB was purified. *V.cholerae recA* was extracted from pTOPO-RecA*_Vch_* by EcoRI digestion and cloned in the same restriction site in vector pGB. **p4640** (pSW29tcat::IntI4_Rbs_GFP) plasmid carrying the *intIA_vch_-gfp* transcriptional fusion:

A pSW23T-aph derivative (plasmid p4730) (Guerin et al., 2009) was used as vector. *gfp* from plasmid p8264 was amplified using oligonucleotides 1049/1050 containing an *rbs*. *intIA* was amplified from N16961 using oligonucleotides 1047 (complementary to 1050)/1048. The two fragments were than fused using assembly PCR (1047/1050), the assembled fragment was BamHI+PstI digested and cloned in p4730 digested with the same enzymes, yielding plasmid p4640. Plasmid p4640 was delivered from *E.coli* β2163 by conjugation and recombined into *V.cholerae* strains by homologous recombination following a protocol previously described (Guerin et al., 2009). Allele integration in *ΔrecA* strains was accomplished through temporary RecA complementation using an exogenous *recA* gene carried on a thermosensitive plasmid (pJJC2791, (Cronan, 2003)).

The different constructions and mutations were introduced in the chromosomes of *V.cholerae* by homologous recombination of suicide plasmids, mainly pSW23T derivatives unless specified otherwise. All constructs were delivered from *E. coli* β2163 (or β3914 for plasmids derived from pSW7848 (pSW4426T (Le Roux et al., 2007; Val et al., 2008) expressing *ccdB*) by conjugation following a protocol previously described (Demarre et al., 2005; Val et al., 2008). All PCR amplifications for gene cloning were carried out with the Herculase II polymerase. (Stratagene). *recA* deletions in the *E.coli* chromosome were introduced by classical techniques of P1 transduction (Miller J, 1992).

**Strain 7949**: The *attC_aadA7_* site, and 130bp upstream region, were amplified from the strain w6851 using oligonucleotides 299 and 396. The *dapA* gene was amplified from MG1655 using oligonucleotides 399 and 353, and both PCR fragments were assembled through fusion PCR. The PCR product was cloned into pSW23t_*attP* (w4849) (Bikard & al., 2010) between EcoRI and BamHI.  The *attI1* sites and the BBa_J23100 promoter  were successively added upstream of the *attC* site by subcloning between the EcoRI/XbaI digested plasmid and EcorI/SpeI digested inserts. The *attI1* site is BBa_J99002 from the registry of standard biological parts (Bikard & al., 2010), and BBa_J23100 is a constitutive promoter from the registry. The resulting plasmid p7923 was integrated into the *attB* site of the ω7784 chromosome through lambda recombination mediated by plasmid pTSA29-CXI (Valens & al., 2004). w7784 is MG1655 deleted for the *dapA* gene following the method described by Chaveroche et al., 2000.

**Selection of conjugants and total number of recipients in conjugation assays:** The total number of recipients was obtained by counting blue cells in LB-Xgal plates for E.*coli* 7651, and LB-Xgal- km for V.*cholerae* 7453 (donor DH5α is deleted for *lacZ* and forms white colonies). The number of conjugants was obtained by counting blue cells on LB-Xgal-Ap for RP4 and R6Kdrd conjugation in E.*coli* 7651, on LB-Xgal-Trim for R388 x E.*coli* 7651, LB-Xgal- tc for R64drd x E.*coli* 7651; LB- km -ap for RP4 x V.*cholerae* 7453, LB- km -Ap-Xgal for R6Kdrd x V.*cholerae* 7453, LB- km -Trim for R388 x V.*cholerae* 7453 and LB- km - tc for R64drd x V.*cholerae* 7453.

Antibiotics were used as follows : 100µg/µl Ap, 50µg/µl km , 10 µg/µl tc , 25µg/µl Cm for *E.coli* and 5µg/µl Cm for *V.cholerae*.

**β-galactosidase tests**: (Miller, 1992) 2 ml of resuspended cells were lysed with 50µl chloroform and 50µl SDS 0.1%. After 5min incubation at room temperature, 1.5ml Z-buffer (8.52g/L Na_2_HPO_4_, 5.5g/L NaH_2_PO_4_, 0.750g/L KCL, 0.25 g/L MgSO_4_ with 7µl/ml β-mercaptoethanol added extemporaneously) was mixed with 500µl of sample and incubated 5 min at 37°C. 500µl of ONPG (o-nitrophenyl-β-D-galactopyranoside, 4mg/ml) were than added to allow the β-galactosidase reaction to turn colorless ONPG to yellow ONP. As soon as the yellow coloration appeared, 500µl of Na_2_CO_3_ (1M) was added to stop the reaction and the reaction time was measured. According to the Miller formula (J. Miller, 1972):

Units at time Tx = 1000*OD_420nm_/ε*final volume/reaction volume*2.5/sample volume*10^6^*1/reaction time minutes, where ε is the molar extinction coefficient (5.10^3^ / mol ONP). So **units at time** **t_x_ = 1000*OD_420nm_ *1.4*1/time**

And specific activity = [measured units at time Tx]/[dry weight] where dry weight= 0.25mg/ml when OD_600nm_=0.71, which is an estimation of the quantity of bacteria. Instead of using an estimation based on the OD_600nm_, we counted the actual number of *cfu* as described above and used the following formula :

**specific activity for total population = [measured units t_x_] / [nb of recipient]**

**specific activity per conjugant = [****measured units at t_x_ –** **(units at t_0_ x nb of recipient cells at t_x_) ] / nb of conjugants at t_x_,** where (units at t_0_ x nb of recipient cells at t_x_) is the basal expression per cell when SOS is not induced. Each assay was reproduced at least 5 times.

**SXT conjugation** : Overnight cultures of donor and recipient strains were washed 3 times in LB and were mixed at a 1:1 ratio. SXT transfer was induced by adding MMC (0.2µg/ml) in the overnight culture. Conjugations were performed as described for R plasmids, for 0 to 6 hours for β-galactosidase tests and overnight for FACS analysis.

**Southern blot:** Oligonucleotides’ labelling : For cassette displacement profiles a mix oligonucleotides 896 to 905 was used. Probes were 5’-labelled with γ32P -ATP using the following protocol : 1µl of 10 µM oligonucleotide mix was labelled using 1µl Poly-nucleotide-kinase (Fermentas) and 500 µCi of γ32P –ATP in a final volume of 5µl at 37°C for 45min and 68°C for 10 min. Labelled oligonucleotides were purified using the Microspin G25 kit (GE healthcare).

Genomic DNA was purified from 10 ml O/N cultures using Qiagen Blood and tissue Kit. Digestions were performed O/N and migrated on agarose 1 % gels. The gels were dried for 1 hour at 60°C. After denaturation for 20 min in [NaOH 0.5 M ; NaCl 0.15 M] and neutralisation for 20 min in [Tris 0.5 M- pH8 ; NaCl 0.15 M] the gel was incubated 2 hours at 55°C for prehybridization in Rapid-Hyb Buffer (GE healthcare). γ-ATP labelled oligonucleotides were then added and hybridization was done at 55°C for 15 hours. The gel was than washed 3 times in [30% SSC ; 1% SDS] during 45 min at 50°C. DNA bands were revealed by O/N exposition on Amersham hyperfilm.

**Flow cytometry analysis:** The percentage of cells expressing gfp was determined by taking the uninduced cells (*V. cholerae* mixed with plasmidless *E. coli*) as negative reference and cells constitutively expressing GFP as positive control. Figure S3 shows an example.

**Cassette excision measurements and selection**: The recipient strain was *E.coli* 7949 p7755. This strain is km -resistant and carries an insertion in the *dap*A gene, thus needs DAP in the medium to grow, unless excision takes place yielding *dap*+ cells. p7755 is Ap-resistant and contains *intI1* under the control of the natural *lexA box* promoter. In the case of RP4, the recipient contained pTOPO-aadA7 (SpecR) plasmid used as marker for selection purposes (to counter select the donor which carries ApR KmR RP4, thus the same resistances as the recipient). The donor strain was DH5α. Conjugations were performed as described for 4 hours on plates containing DAP. Filters were resuspended in 5ml LB and several dilutions were plated on LB + km or LB + ap medium (LB + spec for RP4) without DAP, to select only recipient cells (AP-resistant or km -resistant), that have undergone recombination (able to grow without DAP).

**Supplemental references**

Avila, P., and de la Cruz, F. (1988). Physical and genetic map of the IncW plasmid R388. Plasmid *20*, 155-157.

Burrus, V., and Waldor, M.K. (2003). Control of SXT integration and excision. J Bacteriol *185*, 5045-5054.

Chabbert, Y.A., Scavizzi, M.R., Witchitz, J.L., Gerbaud, G.R., and Bouanchaud, D.H. (1972). Incompatibility groups and the classification of fi - resistance factors. J Bacteriol *112*, 666-675.

Coetzee, J.N. (1974). Properties of proteus and providence strains harbouring recombinant plasmids between P-lac Rldrd19 or R447b. J Gen Microbiol *80*, 119-130.

Cronan, J.E. (2003). Cosmid-based system for transient expression and absolute off-to-on transcriptional control of Escherichia coli genes. J Bacteriol *185*, 6522-6529.

Demarre, G., Guerout, A.M., Matsumoto-Mashimo, C., Rowe-Magnus, D.A., Marliere, P., and Mazel, D. (2005). A new family of mobilizable suicide plasmids based on broad host range R388 plasmid (IncW) and RP4 plasmid (IncPalpha) conjugative machineries and their cognate Escherichia coli host strains. Res Microbiol *156*, 245-255.

Furuichi, T., Komano, T., and Nisioka, T. (1984). Physical and genetic analyses of the Inc-I alpha plasmid R64. J Bacteriol *158*, 997-1004.

Guerin, E., Cambray, G., Sanchez-Alberola, N., Campoy, S., Erill, I., Da Re, S., Gonzalez-Zorn, B., Barbe, J., Ploy, M.C., and Mazel, D. (2009). The SOS response controls integron recombination. Science *324*, 1034.

Kontomichalou, P., Mitani, M., and Clowes, R.C. (1970). Circular R-factor molecules controlling penicillinase synthesis, replicating in Escherichia coli under either relaxed or stringent control. J Bacteriol *104*, 34-44.

Le Roux, F., Binesse, J., Saulnier, D., and Mazel, D. (2007). Construction of a Vibrio splendidus mutant lacking the metalloprotease gene vsm by use of a novel counterselectable suicide vector. Appl Environ Microbiol *73*, 777-784.

Miller (1992). A short course in bacterial genetics. Cold Spring Harbor, NY: Cold Spring Harbor Laboratory Press.

Miller J, H. (1992). A Short Course in Bacterial Genetics.

Saunders, J.R., and Grinsted, J. (1972). Properties of RP4, an R factor which originated in Pseudomonas aeruginosa S8. J Bacteriol *112*, 690-696.

Val, M.E., Kennedy, S.P., El Karoui, M., Bonne, L., Chevalier, F., and Barre, F.X. (2008). FtsK-dependent dimer resolution on multiple chromosomes in the pathogen Vibrio cholerae. PLoS Genet *4*, e1000201.
